# Laparoscopic Excision of Coexisting Left Tubal and Right Pseudotubal Pregnancy after Conservative Management of Previous Ectopic Pregnancy with Methotrexate: An Unusual Clinical Entity

**DOI:** 10.1155/2015/645826

**Published:** 2015-02-24

**Authors:** Panayotis Xiromeritis, Chrysoula Margioula-Siarkou, Dimosthenis Miliaras, Ioannis Kalogiannidis

**Affiliations:** Aristotle University of Thessaloniki, Falireos 16, 56224 Thessalonik, Greece

## Abstract

Tubal pregnancy concerns 97% of all ectopic pregnancies. Treatment can be either surgical (salpingostomy or salpingectomy) or medical (methotrexate administration). We present a case of a pseudotubal pregnancy after methotrexate treatment of a previous ectopic pregnancy. A37-year-old woman was diagnosed with ectopic pregnancy in the left Fallopian tube. A year ago, she had an ectopic pregnancy in the right tube, which was successfully treated with intramuscular methotrexate. During laparoscopy, two tubal masses were revealed, one in each Fallopian tube, and bilateral salpingectomy was performed. Histological analysis confirmed tubal pregnancy in the left Fallopian tube and presence of endosalpingitis in the right tube with no signs of chorionic villi. The optimal management of such cases has not yet been clarified. However, evaluation of tubal patency after a medically treated ectopic pregnancy would permit proper counsel of the patient on her fertility options, in order to choose the appropriate method of conception to achieve and accomplish a future pregnancy.

## 1. Introduction

Ectopic pregnancy is characterized by implantation of the fertilized ovum in another position but uterine cavity. It concerns 2% of all pregnancies and in 97% of those cases the abnormal implantation occurs in Fallopian tube [1, 2]. The incidence of ectopic pregnancy has been increased over time and risk of recurrence augments proportionally to the number of previous ectopic pregnancies. Sivalingam et al. report a 5–20% risk for ectopic pregnancy after a previous one, whereas the relative risk after two or more previous ectopic pregnancies exceeds 32% [2, 3]. Despite the great medical progress, ectopic pregnancy still remains a predominant cause of maternal death during the first gestational trimester [4]. Diagnosis is based on medical history, ultrasound imaging, and successive measurements of serum beta-human chorionic gonadotropin (*β*-hCG) levels.

Treatment of an ectopic pregnancy can be either medical or surgical and it is individualized according to specific criteria, such as clinical symptoms, maternal health status, ultrasound scan findings, *β*-hCG concentration, and attempt to preserve fertility [3]. Laparoscopic salpingectomy or salpingostomy is considered to be the optimal surgical therapeutic option, while medical management includes administration of methotrexate (MTX) [5]. Approximately 25–30% of ectopic pregnancy cases are appropriate to receive medical therapy, with successful results in a percentage of 78–96%, as mentioned by the Practice Committee of the American Society for Reproductive Medicine [6, 7].

We describe the successful laparoscopic treatment of coexisting right tubal and left pseudotubal pregnancy following a previous one which had been successfully treated with methotrexate.

## 2. Case Report

**Figure 1:**
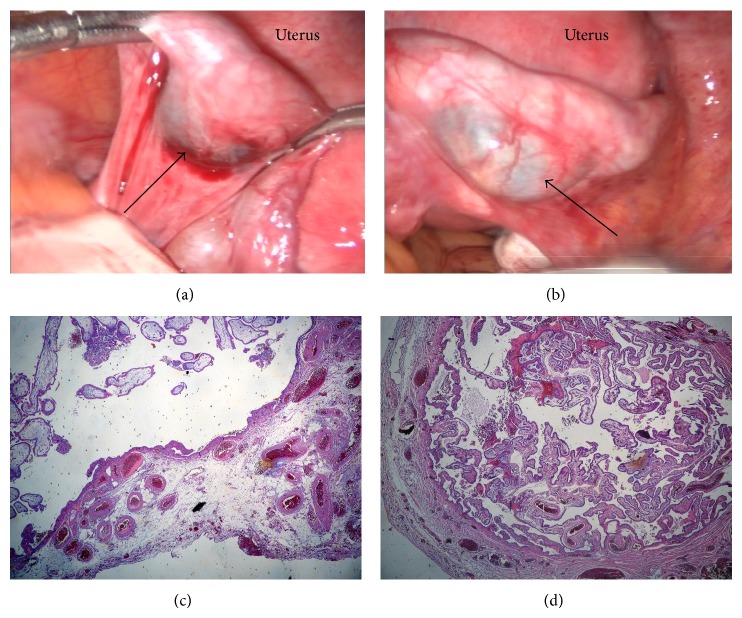
(a) Laparoscopic view of the left Fallopian tube, containing the ectopic pregnancy (arrow). (b) Laparoscopic view of the right Fallopian tube, presenting a congestive dilatation mimicking an ectopic pregnancy (arrow). (c) Histological section of the left Fallopian tube which is distended and contains many chorionic villi (upper left part of the image), confirming extrauterine tubal pregnancy (hematoxylin and eosin stain; original magnification 25x). (d) Histological section of the right Fallopian tube which is distended by an oedematous mucosa with congestive blood vessels, with no chorionic villi (hematoxylin and eosin stain; original magnification 25x).

A 37-year-old nulliparous woman presented with brown spotting and positive pregnancy test. A year ago, she was diagnosed with an ectopic pregnancy in the right Fallopian tube and was successfully treated with a single dose of intramuscular methotrexate (1 mgr/kgr). Clinical examination revealed painful left adnexa. The serum *β*-hCG was stabilized around 300 UI/L at 42 days of amenorrhea, after a brief peak at 580 IU/L. Transvaginal ultrasound scan showed endometrial thickness of 11 mm with no sign of embryonic sac and left paraovarian mass of 12 mm, while pouch of Douglas was free of fluid. Diagnostic laparoscopy was planned due to the diagnosis of extrauterine pregnancy. During laparoscopy, both Fallopian tubes presented an erythemic ampullary dilatation, giving the impression of bilateral tubal pregnancy. Laparoscopic bilateral salpingectomy was performed. Histological analysis confirmed tubal pregnancy in the left Fallopian tube and presence of endosalpingitis in the right tube with no signs of chorionic villi (Figure 1). The presence of endosalpingitis was not extended to the total of Fallopian tube but there was a normal part of Fallopian tube, therefore excluding the possibility that endosalpingitis was caused because of a chronic pelvic inflammatory disease.

## 3. Discussion

Management of a tubal pregnancy is a real challenge, especially when fertility preservation is a major priority for the patient. Even though conservative management is an option, the decision between surgical and medical treatment should be made carefully. At first sight, medical treatment may seem more beneficial compared to surgical treatment, as it is not invasive and maintains the patient's anatomy. However, surgical treatment offers the advantage of direct inspection of the uterus, adnexa, and pelvis in general. Therefore, it allows better assessment of the likelihood of a future pregnancy. Furthermore a number of medically managed patients may require subsequent surgery due to treatment failure [8, 9]. In this case, we performed laparoscopy in order to treat the subsequent contralateral ectopic pregnancy settled down at the left Fallopian tube. Surprisingly, we found two tubal masses: one in the left Fallopian tube, as expected, and the other in the contralateral tube, where the previous ectopic pregnancy was detected one year ago and treated with methotrexate. According to histological examination, this mass, giving the impression of a second concurrent tubal pregnancy, consisted of inflammatory tissue. Although medical treatment is supposed to be the option having the least impact on Fallopian tube function, recent studies in rats contradict the former opinion. Yang et al. support that methotrexate impairs morphology of the Fallopian tubes and causes irreversible damage to their steroid-hormone receptors. In addition, methotrexate was found to inhibit spontaneous isthmus contractions, contributing to a subsequent tubal pregnancy [10–12]. Elito Jr. et al. also mentioned that the effect of methotrexate on rapidly divided cells might result in residual lesion to the Fallopian tube [13].

As was outlined above, both surgical treatment and medical treatment of an ectopic tubal pregnancy may impair Fallopian tube anatomy and consequently future fertility.

Therefore, it gives more information about the probability of future pregnancy. Grau et al. examined the Fallopian tubes of women with ectopic pregnancies that received medical therapy. For this purpose, hysterosalpingography (HSG) was performed 3 months after methotrexate administration. According to their findings, unilateral tubal obstruction was presented in 18.8% of cases and bilateral obstruction in 2.8%, while in 6.3% of women tubal patency was confirmed but with alterations. The authors also proceeded to the study of subsequent pregnancy outcome by the finding of HSG. They found in their study that the possibility of future gestation is 90.6% of women with normal HSG, 76% of those with unilateral tubal permeability. None of the women with bilateral tubal obstruction achieved pregnancy, while subsequent pregnancy percentage in cases with tubal patency with defects slightly exceeded 60%. These results confirm adverse impact of an ectopic pregnancy in future fertility, as in many cases Fallopian tube never returns to its natural morphological status, according to the former HSG findings. This study is pointing out another issue; that is, the identified alterations in tubes might have preexisted and may have caused the ectopic implantation [9].

The pseudotubal ectopic pregnancy case we presented indicates a lack in literature concerning the optimal management of an ectopic pregnancy after methotrexate administration. As fertility is decreased following an abnormal implantation and the most predictive factor of a future gestation is tubal health status, evaluation of tubal patency is critical after an ectopic pregnancy [5]. In particular, in cases where a subsequent gestation is desired, assessment of tubal permeability after medical treatment would permit proper counseling of the patient about her fertility options, in order to choose the appropriate method of conception to achieve and accomplish a future pregnancy.
